# Substrate type and CO_2_ addition significantly influence succinic acid production of *Basfia succiniciproducens*

**DOI:** 10.1007/s10529-023-03406-7

**Published:** 2023-07-03

**Authors:** Márta Balázs, Hunor Bartos, Szabolcs Lányi, Zsolt Bodor, Ildikó Miklóssy

**Affiliations:** 1 Faculty of Science, University of Pécs, Ifjúság 6, 7624 Pécs, Hungary; 2 Department of Bioengineering, Sapientia Hungarian University of Transylvania, Piata Libertatii, 530104 Miercurea Ciuc, Romania; 3 Institute for Research and Development of Hunting and Mountain Resources, St. Progresului 35B, 530240 Miercurea Ciuc, Romania

**Keywords:** *Basfia succiniciproducens*, Fermentation, Gene expression, Substrate utilization, Succinic acid, Systems biology

## Abstract

Metabolic engineering has shown that optimizing metabolic pathways’ fluxes for industrial purposes requires a methodical approach. Accordingly, in this study, in silico metabolic modeling was utilized to characterize the lesser-known strain *Basfia succiniciproducens* under different environmental conditions, followed by the use of industrially relevant substrates for succinic acid synthesis. Based on RT-qPCR carried out in flask experiments, we discovered a relatively large difference in the expression levels of *ldhA* gene compared to glucose in both xylose and glycerol cultures. In bioreactor-scale fermentations, the impact of different gas phases (CO_2_, CO_2_/AIR) on biomass yield, substrate consumption, and metabolite profiles was also investigated. In the case of glycerol, the addition of CO_2_ increased biomass as well as target product formation, while using CO_2_/AIR gas phase resulted in higher target product yield (0.184 mM⋅mM^−1^). In case of xylose, using CO_2_ alone would result in higher succinic acid production (0.277 mM⋅mM^−1^). The promising rumen bacteria, *B. succiniciproducens*, has shown to be suitable for succinic acid production from both xylose and glycerol. As a result, our findings present new opportunities for broadening the range of raw materials used in this significant biochemical process. Our study also sheds light on fermentation parameter optimization for this strain, namely that, CO_2_/AIR supply has a positive effect on target product formation.

## Introduction

Succinic acid, a platform chemical firstly extracted from amber, was described in the sixteenth century (Stellmacher 2014) and nowadays is one of the key building-block chemicals widely used in different industries (Lee et al. 2010; Yin et al. 2015; Ahn et al. 2016; Cimini et al. 2016). Among the chemical methods the hydrogenation process is one of the major technologies for producing succinic acid. Using this technology, it is shown high yield and pure succinic acid can be produced, but processing is expensive, complex and harmful to the environment (Cheng et al. 2012). On the other hand, biological transformation as a way of converting biomass to higher value chemicals is characterized by high yield and selectivity and minimal waste (Cheng et al. 2012; Barcelos et al. 2020). Bio-based succinic acid production potential has already been analyzed and optimized or even commercialized in case of bacteria such as *S. cerevisiae*, *Pichia kudriavzevii*, *E. coli*, *M. succiniciproducens*, *B. succiniciproducens*, *A. succinogenes*, and *Corynebacterium glutamicum* (Schellenberger et al. 2011). Metabolic engineering strategies are commonly used in strain design to convert renewable biomass and different carbon sources into an added-value industrial product (Lee et al. 2002, 2010; Dale 2003). According to the US Department of Energy, succinic acid was recently indicated as one of the top value-added platform chemicals of fermentation (Zeikus et al. 1999; Lee et al. 2002) and currently, the biotechnological production of succinic acid is under continuous optimization in light of research and development activities (Cheng et al. 2012). The development of green technology is a daily challenge due to the need to reduce the pollution caused by the petrochemical industry (Zeikus et al. 1999). Biotechnology throughout metabolic engineering and systems biology has the possibility to create new metabolic pathways and optimize the existing ones to produce chemicals whose microbial production has so far had only a modest industrial significance. In modern biotechnology the in silico methods are unavoidable, hence, the metabolic genome-scale simulation is essential to identify, design and optimize the target product biosynthetic pathways (Lee et al. 2012). Since succinic acid is a key metabolite, many researches in recent years have focused on identifying microorganisms that have increased metabolic potential to produce succinic acid from different carbon sources (Lange et al. 2017; D’Ambrosio et al. 2021). Therefore two main strategies were identified, the first is the isolation of microorganisms with natural potential (Zeikus et al. 1999; Lange et al. 2017), while the second is recombinant systems, well known to the industry (*E. coli, S. cerevisiae*) (Ma et al. 2010; Stellmacher 2014). A native producer of succinic acid *Anaerobiospirillum succiniciproducens*, is capable of producing significant amounts of succinic acid from various carbohydrates, and it is considered as one of the most important succinic acid producers (Lee et al. 2001, 2003). According to the literature, similar metabolic capabilities were observed for *Actinobacillus succinogenes* and *Mannheimia succiniciproducens* as well (Lee et al. 2002). One of the most promising natural succinic acid producers in the family of *Pasteurellaceae* is *Basfia succiniciproducens (B. succiniciproducens)*, which is a facultative anaerobic and capnophilic rumen bacterium isolated from a Holstein cow (Fabarius 2018). In contrast to other *Pasteurellaceae*, *B. succiniciproducens* shows genetic similarity to the patented strain *Mannheimia succiniciproducens* MBEL55E (Stellmacher 2014). When grown in anaerobic conditions, it uses fumarate as the final electron acceptor and produces succinic acid, while fixing CO_2_. It has also been shown the role of CO_2_ as co-substrate during succinic acid production, primarily in case of strains with increased biosynthetic potential (Srinivasan 2012; Song et al. 2007; Lee et al. 2012; Kuenz et al. 2020) (Table 1). Under oxygen limited conditions phosphoenolpyruvate carboxylase is an essential anaplerotic enzyme which catalyzes the CO_2_ fixation to produce oxaloacetate or pyruvate from phosphoenolpyruvate. Oxaloacetate is a building block and a key intermediate molecule in the tricarboxylic acid cycle (TCA) cycle to form succinic acid through malate and fumarate (Becker et al. 2007).

**Table 1:** Engineering and fermentation strategies

Strain	Carbon source (g/L)	Process	Succinic acid (g/L)	Yp/s (g/g)	References
DD1	20 g glucose L^−1^	Batch 0.3 L, pH 6.5, 39 °C, 500 rpm, 0.25 vvm CO_2_	12.3	0.7	Scholten and Dägele (2008)
DD1	Crude glycerol (ecoMotion, GmbH Sternberg)	Chemostat 0.3 L, pH 6.5, 500 rpm, 0.1 L/min CO_2_, D 0.018	5.21	1.02	Scholten et al. (2009)
DD1 (Kuhnert et al. 2010)	50 g glucose L^−1^	30-mL serum bottles, filled with 10 ml of medium with CO_2_ head overpressure	20	0.49	Becker et al. (2013)
BPP7	*Populus nigra* (18–20 g glucose L^−1^ + 2–3 g xylose L^−1^)	Batch 2.4 L, pH 6.5, 0.5 vvm CO_2_	15–18	0.75	Pennacchio et al. (2018)
DSM22022	SSL (spent sulphite liquor)	Continuous fermentation, 1 L bench-top bioreactor. 37 °C, 250 rpm	16–22	0.4–0.55	Dimitros et al. (2018)
DSM22022	15 g glucose L^−1^ 2 g xylose L^−1^	Batch 1.5 l, pH 6.7, recirculated biogas, Process duration 12 h	4	0.25	Babaei et al. (2019)
JF4016	25.4–75.9 g glucose L^−1^	Batch 0.5 L, pH 6.7 0.5 vvm CO_2_	26.8	0.53	Stylianou et al. (2020)
DSM22022	CSL	1 L bench-top bioreactor, 37 °C, 100 rpm and 0.5 vvm CO_2_	19	0.62	Eleni et al. (2021)

**Fig. 1:**
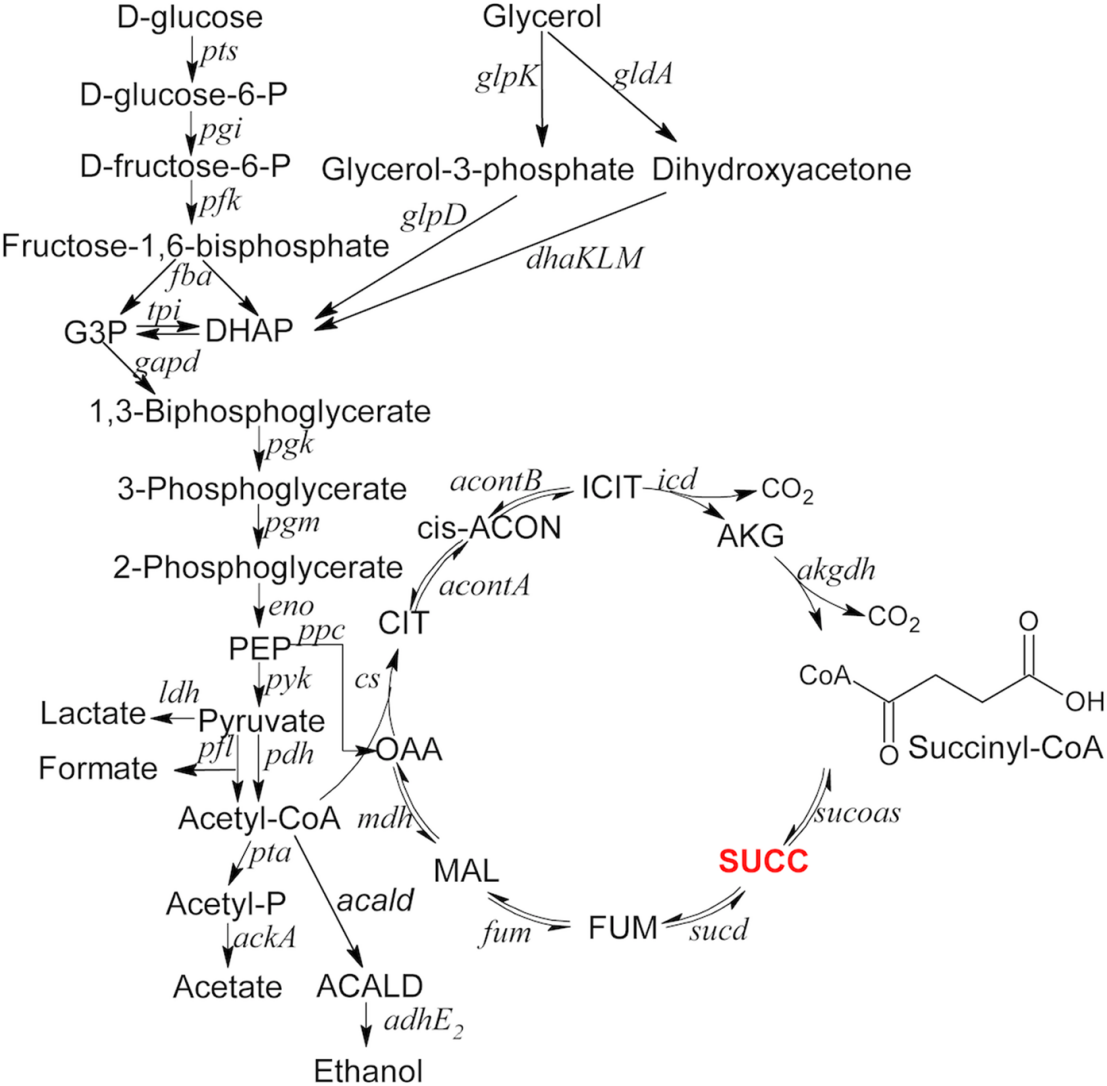
Substrate utilization, organic acid production and TCA cycle metabolic pathways in *B. succiniciproducens* (Sinkler et al.^2019^)

*B. succiniciproducens* has a broad substrate spectrum, which includes glucose, galactose, mannose, sucrose, trehalose and xylose, mannitol, and glycerol (Kuhnert et al. 2010) but due to regional availability and fluctuating raw material costs, research needs to be extended to other carbohydrates (Lange et al. 2017). This valuable platform molecule, as shown on Fig. 1 can be synthesized in reductive and oxidative metabolic pathways of the TCA cycle. The assimilable carbon dioxide can be accessed from metal carbonates (alkali or alkali-earth) or from sparging it directly into the medium during fermentation. The first target substrate has been organic waste which was followed by studies on new bio routes from lignocellulose waste. In the technology of the traditional pulp and paper industry, a significant amount of sugar monomer is produced such as the xylose by-product (Pateraki et al. 2016). Moreover, the growth of the biodiesel industry has derived surplus of glycerol, all of which could be considered as succinic acid production raw materials (Livak and Schmittgen 2001; Lee et al. 2006; Murarka et al. 2008; Yu et al. 2016; Guarnieri et al. 2017; Zhang et al. 2019; Kim et al. 2020).

The objective of the current research was a thorough examination of the biosynthesis of target-metabolite succinic acid in *B. succiniciproducens* based on a systems biology approach. In the framework of this study, we offer a metabolic insight of this valuable strain regarding growth on xylose and glycerol in comparison to glucose, in terms of fermentation, target product concentration and gene expression data. In silico analyses were carried out using the available metabolic model and in order to investigate different substrates the model has been completed with new pathways describing the reactions necessary for glycerol and xylose metabolism. The most important analyses are as follows: flux balance analysis, theoretical maximum of target compound production, and production envelope analyses. In silico studies were followed by wet experiments comprising of determination of culture conditions under different substrates in small volumes, as well as in controlled bioreactor settings.

## Materials and methods

### In silico

In our simulations we used the most recent metabolic model of *B. succiniciproducens* (Becker et al. 2013), which contains more than 60 reactions and 58 metabolites. To increase target compound formation and the ability to degrade other substrates first we added new reactions to the model, namely xylose isomerase (EC 5.3.1.5.), xylulokinase (EC 2.7.2.17), glycerol kinase (EC 2.7.1.30). In silico simulations were carried out by using MATLAB (Mathworks Inc., Natick, MA, USA) and COBRA Toolbox software packages with Gurobi Optimizer (Gurobi Inc.) (Becker et al. 2007; Schellenberger et al. 2011). Substrate uptake rates were fixed to a value according to the literature: glucose = 7.7 mmol⋅g^−1^⋅h^−1^, xylose = 9.24 mmol⋅g^−1^⋅h^−1^and glycerol = 15.4 mmol⋅g^−1^⋅h^−1^. To simulate anaerobic conditions, the oxygen uptake rate was set to zero. Flux balance analysis and production envelope analysis was performed with the modified (updated) metabolic reconstruction.

### Strain

*B. succiniciproducens* wild type (DSM-22022) isolated from cow rumen was purchased from Leibniz Institute DSMZ-German Collection of Microorganisms and Cell Cultures. The cells came in freeze dried form, rehydration was carried out in specified medium TSB, containing 17 g peptone from casein L^−1^, 3 g peptone from soymeal L^−1^, 2.5 g glucose L^−1^, 5 g NaCl L^−1^, 2.5 g K_2_HPO_4_ L^−1^, and pH 7.0. Working cell stocks were maintained on TSB-agar solid media, and cultures were regularly checked for purity. Long-term maintenance was carried out as 25% glycerol stocks at − 80 °C.

### Microplate experiments

The cells were removed from − 80 °C stock and were suspended in TSB broth with 5 g glucose L^−1^ substrate concentration. The preliminary cell growth was carried out at 37 °C and 130 rpm for 8–10 h. Microplate experiments were carried out on M9 minimal medium with three different substrates in different concentrations, and with addition of 1 g yeast extract L^−1^. During fermentation two concentrations were used for each substrate: 5 g and 20 g glucose, glycerol and xylose L^−1^, respectively. Inoculation was set to an initial optical density of ~ 0.3 (λ = 600 nm) in each cell containing well (96-well microplate, BRAND plates^®^, Germany), cultures were set up in triplicates for every experimental condition. Cultures were maintained at 37 °C for 24 h, optical density was determined every 30 min, with 90 s shaking at 150 rpm before each measurement with a FLUOStar Optima microplate reader (BMG Labtech GmbH, Germany).

#### Flask experiments

Cells pre-cultivated in TSB broth with 5 g glucose L^−1^ substrate concentration at 37 °C and 130 rpm conditions, were centrifuged. Supernatant was discarded and the initial cell concentration was set to ~ 0.3 (λ = 600 nm) in 250 mL Erlenmeyer flasks and the fermentations were carried out in M9 minimal medium at 37 °C and 130 rpm.

### RNA extraction and gene expression analysis

#### RNA isolation

Samples for RT-qPCR analysis were taken from exponential phase (OD600 ~ 1) cultures from flask experiments. RNA isolation was performed using the GeneJET RNA Purification Kit (Thermo Scientific) following the manufacturer's instructions. The concentration of total RNA obtained was determined spectrophotometrically (GenWay, Genova Nano). The purified RNA was stored until further use at − 80 °C.

#### RT-qPCR

The housekeeping gene 16SrDNA was chosen as a reference gene for relative gene expression assessment (Kim et al. 2020). Target-specific forward and reverse oligonucleotides are described in Table 2.

**Table 2:** Oligonucleotides used throughout the study

fruA5 F	5′-GAGATAAACAAAGGCTTTATGCTTGATTAGGAAATTGTTTTACTGCGGCAGCCAAAACCTGGT-3′	Lange et al. (2017)
fruA6 R	5′-CCGCTCGAGTAGGAGTAACTCAAGGTCACCGTTTG-3′	Lange et al. (2017)
rbsK1 F	5′-GCGCCATGAAAAAGCTAACTGTTCTCG-3′	Genentech
rbsK2 R	5′-CCGCGCTGTTGTTCATTAAGAAAGGCTAAT-3′	Genentech
ldhA1 F	5′-ATGAAAGTTGCCGTTTACAGTAC-3′	Genentech
ldhA2 R	5′-TTAACCTTCAACGCTATTTTCGC-3′	Genentech
pfl1 F	5′-ATGGCTGAATTAACAGAAGCTC-3′	Genentech
pfl2 R	5′-AGAATAGTTAAGTTTGGTTCC-3′	Genentech
27F	5'-AGAGTTTGATCCTGGCTCAG-3'	Chen et al. (2015)
1492R	5'-GGTTACCTTGTTACGACTT-3	Chen et al. (2015)

RNA-based cDNA synthesis was performed by reverse transcription (RevertAid cDNA Synthesis Kit, Thermo) using Oligo dT primer. Reaction compositions were as follows: 4 µL 5X reaction buffer, 2 µL 10 mM dNTP, 1 µL Oligo dT, 1 µL RNase inhibitor, 5 µg RNase template, Revert Aid M-MuLV RT (200 u⋅µL-1) 1 µL. As a first step, the template was weighed with the Oligo dT primer and the RNase inhibitor, then kept at 65 °C for 10 min and then rapidly placed on ice, then the other reaction components were weighed and incubated at 42 °C for 1 h. The enzyme was inactivated at 95 °C. The concentration of the obtained cDNA was determined spectrophotometrically (GenWay, Genova Nano). The Mx3005P qPCR System (Agilent Technologies, USA) was used to run the two-step RT-qPCR reactions, the cycling profile is presented in Table 3.

**Table 3:** Thermal profile of the qPCR reactions

Method	Steps	Temperature (°C)	Time (min)	-
qPCR	Initial denaturation	95 °C	7 min	1X
	Denaturation	95 °C	30 s	35X
	Annealing/data collection	47 °C	30 s	
	Extension	72 °C	1 min	
	Dissociation/data collection	72 °C	1 min	1X

Fluorescence intensity was measured in end-point mode, after each annealing step of the reactions. Reaction specificity and presence of non-specific reaction products was assessed based on fluorescence data recorded from dissociation curves. Gene expression was quantified by the 2-ΔΔCt method. Comparative quantitation was performed firstly based on amplification plots derived from fluorescence intensity values for the reactions normalized to the reference dye fluorescence intensity (CRX), based on calculated C(T) values. ΔC(T) values were yielded by normalization to the C(T) values displayed by the reference gene.

#### Bioreactor experiments

For inoculum preparation 500 μL of − 80 °C cell stock was transferred to 10 mL previously prepared TSB pre-culture medium containing 5 g substrate L^−1^ and was incubated for 8 h at 37 °C. The next transfer was to 60 mL 5 g glucose L^−1^ containing TSB medium and incubation parameters were the same. The final inoculation culture was prepared in 400 mL 5 g glucose L^−1^ containing TSB with parameters described previously. After the preliminary incubations the cells were centrifuged for 15 min at 1300×g, the cell containing pellet was suspended with saline solution (0.9% NaCl), and 1 L total volume reactors containing 0.5 L M9 media with 20 g substrate L^−1^ were inoculated and the initial optical density was set up to 0.3 at 600 nm. Sartorius Biostat^®^A Plus Bioreactors with BioPAT^®^ MFCS/DA control unit and data collecting unit were used for fermentation. Temperature and pH was set to 37 °C and 7.0, and culture pH was maintained with 1 M NaOH and 1 M HCl. Agitation speed in the bioreactors was set to 150 rpm. Experimental conditions were glucose, xylose or glycerol as substrates and CO_2_ or CO_2_/AIR inlet.

During the first fermentation experiment the reactor headspace was sparged with 60⋅60^–1^ mL⋅min^−1^ CO_2_/AIR^−1^ mixture. The second fermentation operated at anaerobic conditions with exclusively CO_2_ at 60 mL⋅min^−1^ flow rate. Bioreactor experiments were performed in duplicates for each experimental condition, and samples were collected every 2 h for optical density measurements and chromatography analysis.

### Sample analysis

Metabolites were analyzed with high pressure liquid chromatography (Agilent Infinity 1260), equipped with RID and DAD detectors in order to decipher the substrate consumption and the production of organic acids respectively. Samples collected were centrifuged at 16,400×g for 15 min, filtered through a 0.2 µm Whatman filter and stored at − 20 °C until analysis. Determination of substrate, succinic acid, acetic acid, formic acid, lactic acid was carried out using a Coregel 87H3 column at 50 °C, applied pressure 70 bar. As eluent 0.008 N H_2_SO_4_ solution was used at 6 mL·min^−1^ flow rate.

## Results and discussions

According to our objectives, the in silico physiological study was carried out first followed by gene expression and fermentative metabolism analyses under different environmental conditions. Special attention has been paid to the study of carbon sources suitable for industrial application, such as glycerol and xylose, as these have been scarcely studied in the relevant literature. The effect of carbon sources on the growth potential of *B. succiniciproducens* was investigated at different concentrations in small volumes, and then fermentations were performed in the presence of CO_2_. In the fermentation process substrates were added at selected concentrations to determine the metabolic profile (organic acids, substrates) and the expression levels of the genes encoding fructokinase (*rbsK*), phosphotransferase (*fruA*), lactate dehydrogenase (*ldhA*) and pyruvate formate lyase (*pfl*). Gene expression studies can provide valuable information on the mechanism of substrate utilization, the extent of lactate and formate production, and phosphoenolpyruvate-dependent (*fruA*) and independent (*rbsK*) sugar metabolism. Lastly, in bioreactor-scale fermentations, the effect of CO_2_ or CO_2_/AIR inlet was examined regarding substrate consumption and organic acid production.

### In silico prediction of succinic acid production

Based on in silico predictions, we can affirm that xylose was the best substrate for producing succinic acid (5.79 mmol⋅g^−1^⋅h^−1^), while the highest biomass formation was obtained in case of glycerol.

**Fig. 2:**
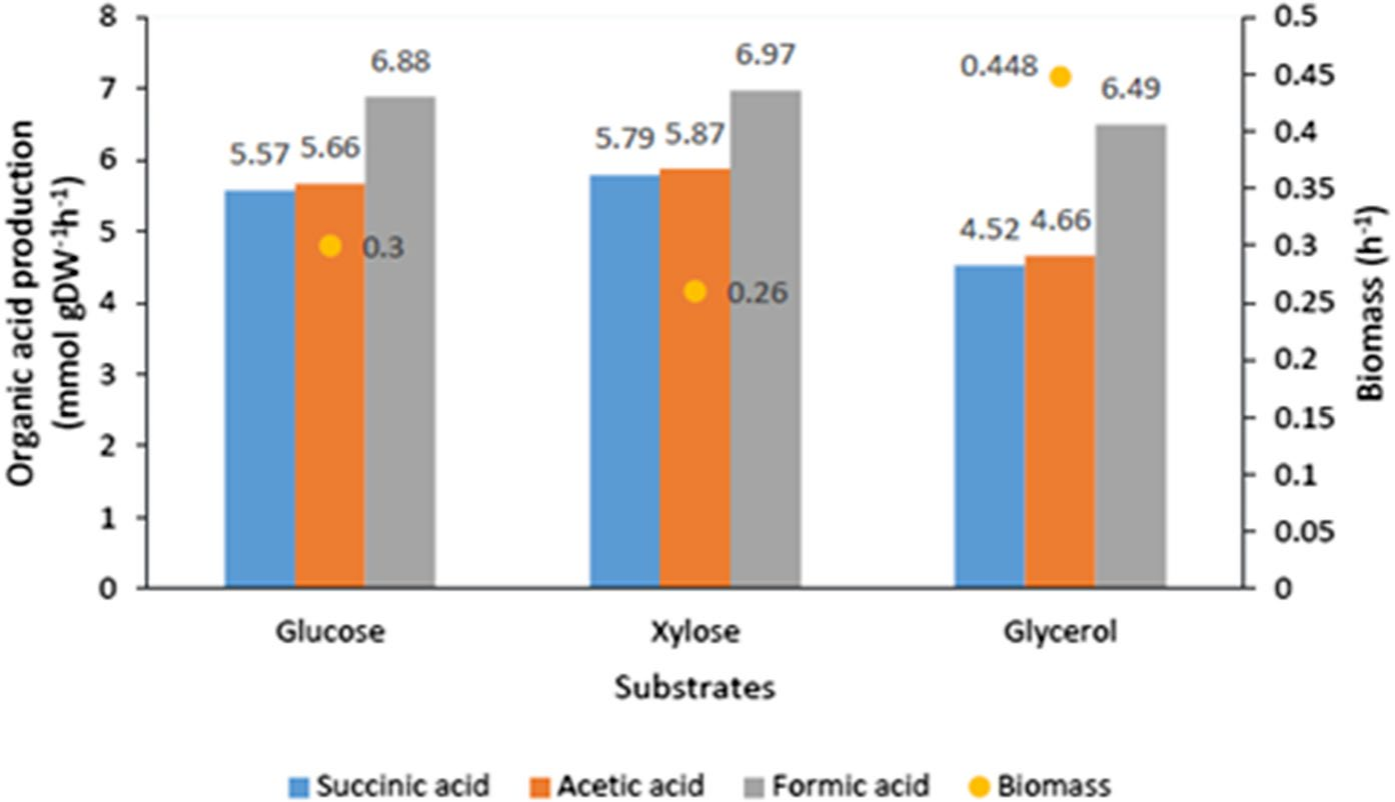
Wild type *B. succiniciproducens* productivity and biomass formation (in silico experiments)

In silico experimental data on Fig. 2 show biomass formation and produced organic acids flux, according to the results, formic- and acetic acid appear to be the most active pathways of organic acid formation. Succinic acid yield relative to dry cell weight does not show significant difference in case of the examined substrates. Similarly, acetic acid production presents similar metabolic pathway fluxes. Metabolic pathways toward formic acid production appear to be the most active, generating the highest flux of organic acid on all three carbon sources.

### Microplate scale population dynamics

**Fig. 3:**
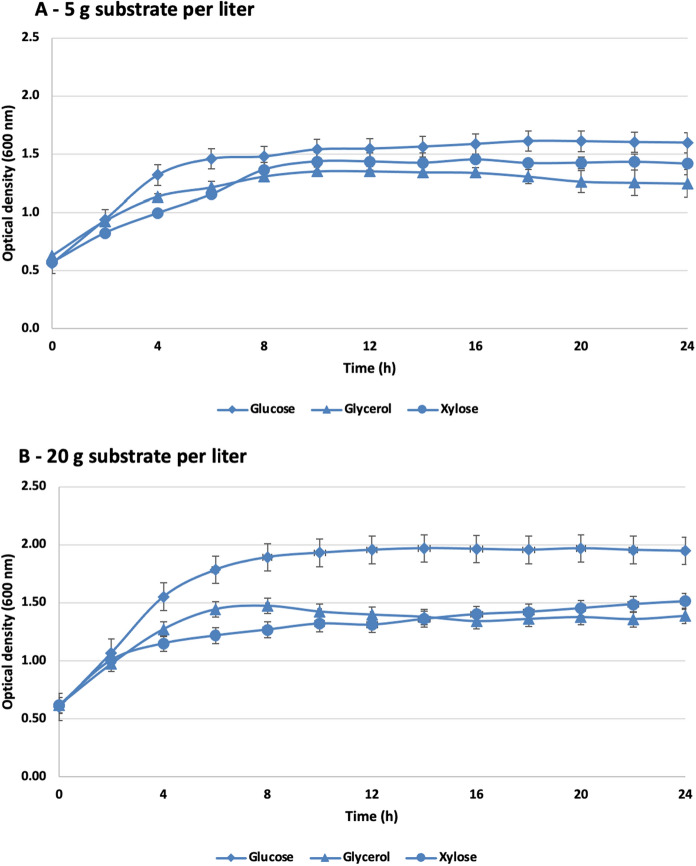
Microplate experiments. OD600 values during growth of *B. succiniciproducens* in presence of different glucose, glycerol and xylose carbon source concentrations in minimal medium are presented. No significant difference can be detected in growth phases or maximal optical density in cultures grown in glucose, glycerol or xylose as the sole carbon sources at 5 g·L^−1^ (**A**) or 20 g·L^−1^ (**B**) concentrations

Three industrially relevant carbohydrates were used in our microplate experiments as sole carbon sources: glucose, glycerol and xylose. After 24 h of fermentation, *B. succiniciproducens* cultures were stable and were able to utilize effectively all applied carbon sources, showing no substrate inhibition under these experimental conditions. Figure 3 illustrates growth curves obtained on different substrates by measuring the change in optical density at 2-h time intervals.

Our experiment was performed in a microtiter plate with a final volume of 200 µL. At both 5 g and 20 g substrate L^−1^ concentration, the population dynamics phases can be well defined. The adaptation phase was practically missing in case of all examined substrates, which can be explained by the fact that the initial high cell number can adapt rapidly in a relatively small volume. As a result, the logarithmic phase begins almost immediately on all substrates and lasts, on average, until the ninth hour of fermentation. In the case of the glycerol and xylose, a lower rising logarithmic phase can be observed. In both cases, we can see that the bacterial cells show higher OD values on glucose, from this, we can draw the conclusion that the bacterial cells were able to utilize glucose more efficiently as a carbon source through their metabolic pathways than glycerol and xylose. In case of 5 g substrate L^−1^ concentration a longer exponential phase was observed, while the higher (20 g substrate L^−1^) concentration resulted in a shorter exponential phase with higher obtained cell density. Based on the series of experiments, it can be affirmed that the cell line produces stable cultures on the tested substrates, no substrate inhibition can be observed in this concentration range, and similar OD values can be achieved by all three applied substrates.

### Gene expression data from flask experiments in CO_2_ atmosphere

**Fig. 4:**
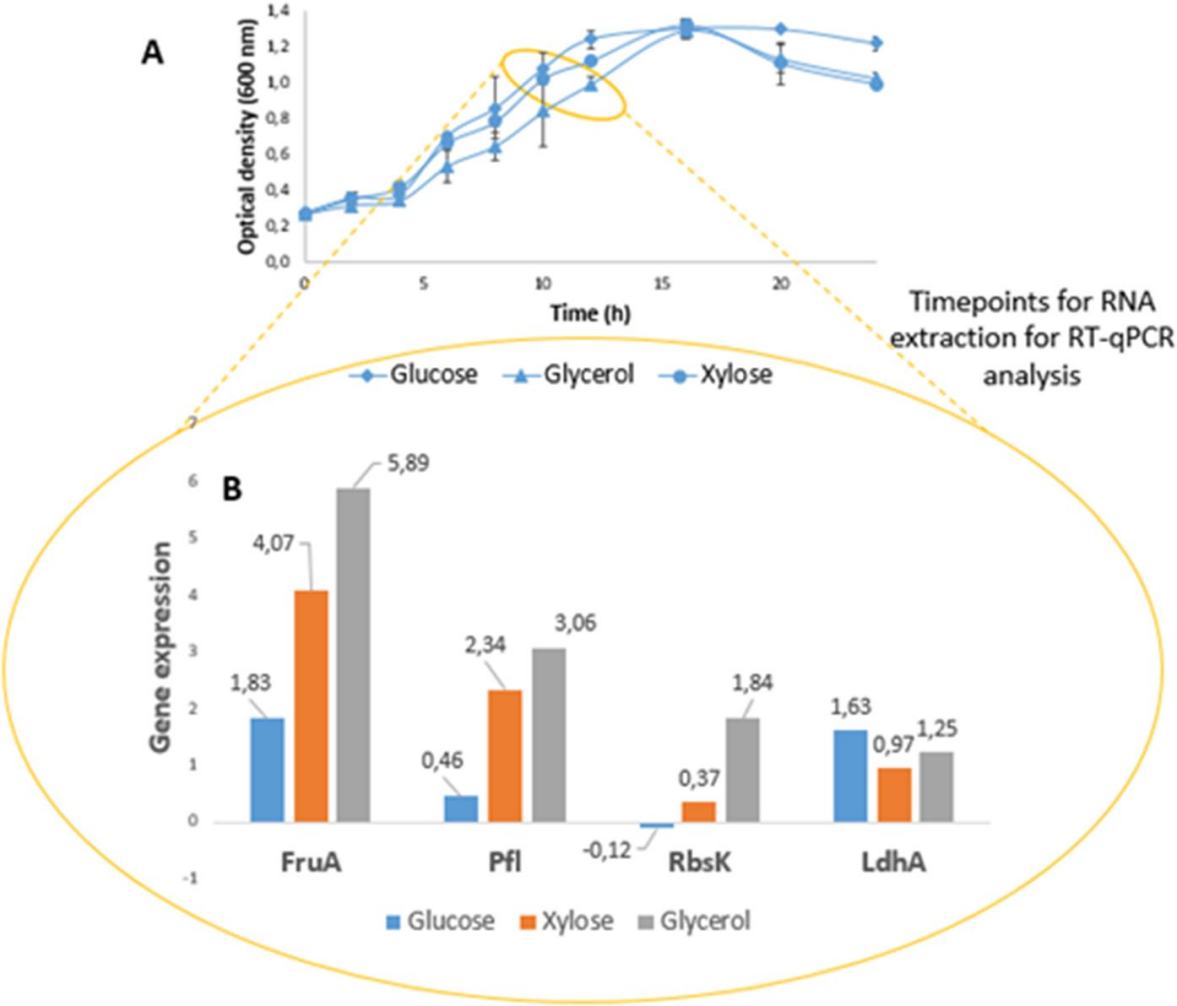
Flask experiments and RT-qPCR results

Population dynamics were further monitored in larger volume fermentations at a substrate concentration of 20 g substrate L^−1^ in the presence of CO_2_. As data on Fig. 4A show, the growth phases are nicely separated, after a slower adaptation phase, similar dynamics characterize the exponential phases of cultures grown on all three substrates. The increase in biomass gave similar results to our experiments on the microtiter plate, and also supported our previous results in the interval of the logarithmic phase.

Quantitative reverse transcription PCR was applied (RT-qPCR) to obtain an insight in the expression of relevant genes of the central carbon metabolism to contour dominant pathways during fermentation and the metabolic pathways of organic acid formation. Figure 4B shows the relative gene expression changes in *B. succiniciproducens* cultures on glycerol and xylose substrates compared to glucose as a result of RT-qPCR reactions with the listed gene- specific primers. 16SrDNA served as a reference gene, while differences in the level of relative gene expression are presented as fold-change values compared to fermentations conducted on glucose carbon source. We can see that the *ldhA* gene expression is the most intense relative to glucose, with a fold-change of ~ 1.5 for both substrates, so lactate production appears to be a major fermentation pathway for organic acid formation. In addition to lactic acid, the second most important production was observed for formic, though to a lesser extent on transcriptional level (0.27 and 0.16 fold-change relative to glucose in case of cultures grown on xylose and glycerol, respectively). From the point of view of the consumption of carbon sources, the expression of the studied *fruA* and *rbsK* genes can provide information based on our experimental conditions. According to our observations, fructose phosphorylation by PEP-independent *rbsK* shows higher expression in the case of xylose, which is an encouraging result in terms of relative target product yield. Selection of the studied genes was carried out based on data on the literature, in order to have an understanding of the expression of *ldhA* and *pflD* genes in relation to carbon flux direction to lactate and formate, and *fruA* and *rbsK* genes in terms of monosaccharide metabolism in case of xylose and glycerol used as sole carbon sources. *LdhA* and *pflD* were formerly targeted for gene deletion for improved succinic acid production from glucose (Becker et al. 2013). The *fruA* and *rbsK* genes are interesting in terms of monosaccharide metabolism in *Basfia succiniciproducens*, also targeted for deletion and overexpression, respectively, in a former study by Lange 2017”.

### Effect of CO_2_ addition on organic acid production in bioreactor cultures

**Fig. 5:**
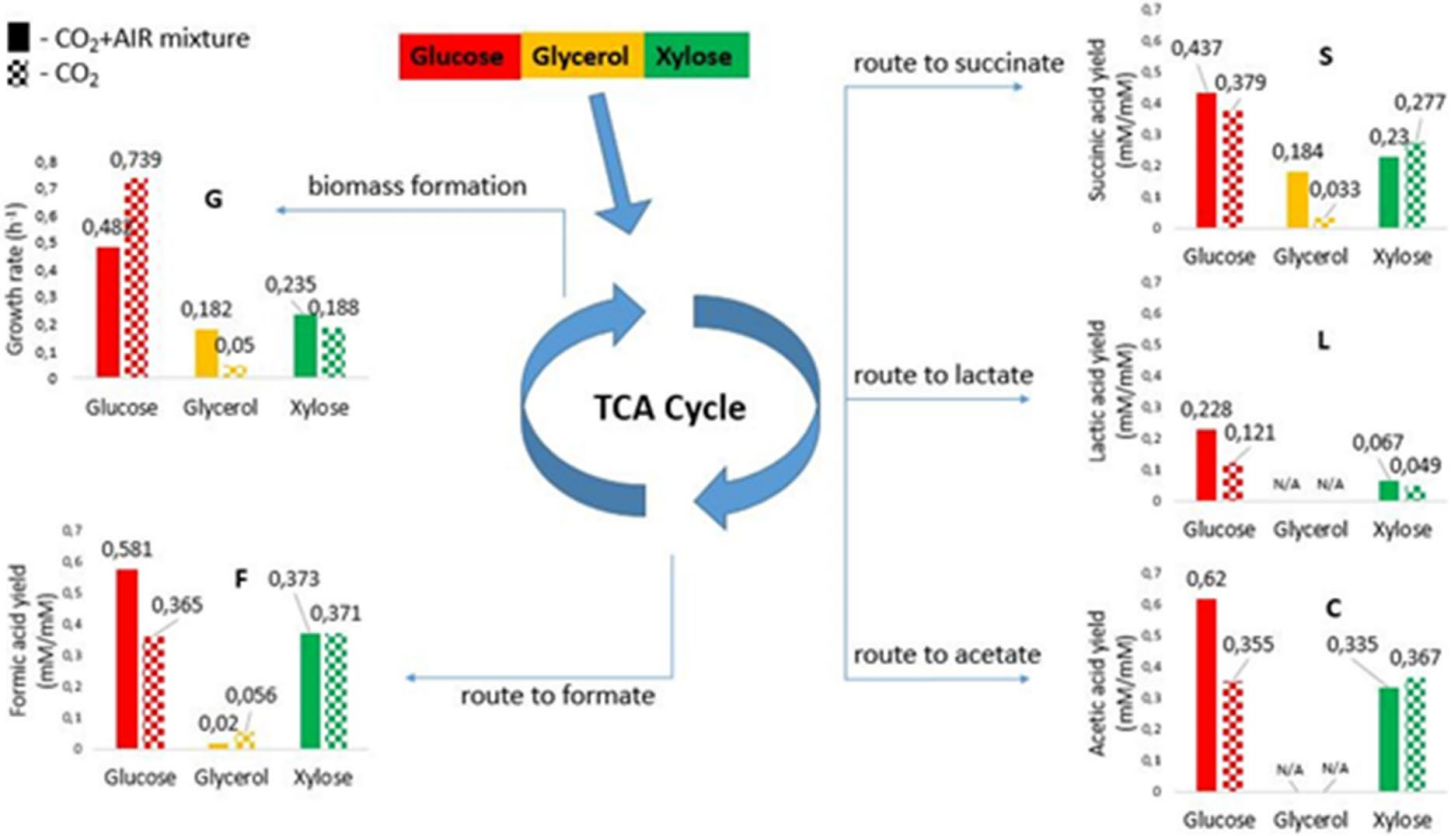
*B. succiniciproducens* metabolic profile and population dynamics on M9 medium. CO_2_/AIR mixture (60/60 ml/min) and CO_2_ (60 ml/min), respectively. G—bacterial growth rate (values calculated to exponential phase), F—formic acid concentration and substrate concentration rate; S—succinic acid concentration and substrate concentration rate; L—lactic acid concentration and substrate concentration rate; C—acetic acid concentration and substrate concentration rate (values calculated to the whole interval (12 h) of fermentation)

The effect of different gas phases (CO_2_ and CO_2_/AIR) on bioreactor-level fermentations was analyzed. The focus was on biomass yield, as well as on substrate consumption properties and metabolite production profiles of the strain, hence, the fermentation was monitored continuously and the samples were analyzed by liquid chromatography (Fig. 5).

The results revealed significant differences related to biomass and succinic acid production in case of glycerol. Since *B. succiniciproducens* is a capnophilic microorganism, CO_2_ can be utilized and may be assimilated, in order to decipher the effect of CO_2_ investigations on biomass and fermentation profile two parallel experiments were carried out in duplicates for each substrate and the bioreactor headspace was sparged with CO_2_/AIR mixture with 60/60 mL min^−1^ flow rate. In order to understand the role of CO_2_ and AIR as well during the second set of experiments the bioreactor gas space was sparged exclusively with CO_2_ with a flowrate of 60 mL min^−1^.

The CO_2_ gas phase applied tries to imitate the original environmental conditions of *B. succiniciproducens*. However, in the case of glycerol as a carbon source, which is more reduced compared to glucose, the biomass production and organic acid production as well can be reduced under strict anaerobic conditions. To overcome this limitation, we used the CO_2_/AIR mixture as an alternative approach. While the examined strain shows optimal growth under microaerobic conditions, the presence of oxygen as final electron acceptor may lead to higher produced biomass quantity and ultimately larger amount of succinic acid. Regarding biomass production, the highest value was 0.739 h^−1^ for glucose in the CO_2_ gas phase, while a similar value was obtained for xylose and glycerol only with CO_2_/AIR gas phase. Based on our findings, we recorded a relevant effect of the applied gas phase on fermentation outcome under different substrate conditions. In the case of glucose used as the sole carbon source, applying only CO_2_ resulted in a higher (0.54 g biomass L^−1^) cell density, compared to CO_2_/AIR conditions where a lower cell concentration (0.38 g biomass L^−1^) was observed. In case of xylose under both aeration techniques (CO_2_/AIR and CO_2_) similar cell concentration was noted (0.72 and 0.71 g biomass L^−1^, respectively). Microaerobic conditions (CO_2_/AIR) seem to be more favorable for the strain in case of glycerol used as the sole carbon source, with a significant—almost triple quantity—increase in biomass production (0.46 g biomass L^−1^) being measured, compared to CO_2_ condition (0.16 g biomass L^−1^). Furthermore, the highest succinic acid yield was obtained on glucose (0.437 mM·mM^−1^), followed by xylose (0.23 mM·mM^−1^) and finally glycerol (0.184 mM·mM^−1^).

The highest organic acid production was recorded in the form of acetic acid and formic acid from glucose and xylose as main carbon sources. The yield of acetic acid on glucose was 0.62 mM·mM^−1^, and similar values were obtained for formic acid (0.581 mM·mM^−1^). Changing the substrate to xylose comparable tendency was recorded, higher acetic acid yields under anaerobic conditions (0.367 mM·mM^−1^) and 0.277 mM·mM^−1^ yields of succinic acid, while formate (0.373 mM·mM^−1^) and lactate (0.067 mM·mM^−1^) yields were higher under aerobic conditions. In the case of xylose, the formation of acetic acid and formic acid can be observed in almost similar amounts, the metabolism of *B. succiniciproducens* under these conditions shifts towards the production of all four monitored organic acids. Consequently, based on both in silico and wet experiment results, these seem to be essential pathways to optimize to increase succinic acid yield. Regarding glycerol as a carbon source, in the aerated gas phase the main flux shifted towards succinic acid at a yield of 0.184 mM·mM^−1^, a little acetate was also produced (0.02 mM·mM^−1^), while in the anaerobic phase, acetic acid was produced in larger amounts.

## Conclusions

During our research, we used both in silico and laboratory approach for monitoring *B. succiniciproducens* culture stability and succinic acid yield on glucose, xylose and glycerol used as carbon sources. The promising rumen bacteria, *B. succiniciproducens* is suitable for improving succinic acid production starting from both xylose and glycerol, our findings thus open up new possibilities to expand the raw material spectrum for this important chemical.

- Three renewable based substrates were investigated, namely glucose, xylose and glycerol, the biological utilization of each having several advantages e.g. renewable, currently appearing as a by-product in various industries, etc. *B. succiniciproducens* was investigated using in silico methods to explore the metabolic potential on these substrates and to find the ideal combinations for future strain optimization. Based on the results of the in silico network analysis, the tested strain is more capable to utilize glucose, and formate, lactate and acetate were predicted as major organic acid products.
- The substrate preference of our *B. succiniciproducens* strain was also studied in laboratory conditions. The highest optical density, thus biomass production was recorded in case of glucose, however, population dynamics showed that the strain produces stable cultures without signs of substrate inhibition in case of xylose and glycerol as well. Moreover, growth rates determined in silico as well as wet experiments showed a good correlation under the given experimental conditions.
- Gene expression profile conducted for several key enzymes of the primary metabolism, resulted in a relatively substantial difference in the case of *ldhA* gene in xylose and glycerol cultures compared to glucose, and a smaller deviation in the case of *rbsK* gene. A comparison of the expression profiles revealed that the strain optimization solution described in the literature for sucrose is a good strategy for the production of succinic acid from xylose carbon source due to the higher *rbsK* and lower *fruA* expression levels.
- Bioreactor-scale fermentations shed light on more suitable culture conditions for this strain, as target product yield and biomass production were improved in CO_2_/AIR atmosphere in case of glycerol compared to culturing solely under CO_2_ gas phase.

## Data Availability

Not applicable.
